# Cannabidiol as a Therapeutic Alternative for Post-traumatic Stress Disorder: From Bench Research to Confirmation in Human Trials

**DOI:** 10.3389/fnins.2018.00502

**Published:** 2018-07-24

**Authors:** Rafael M. Bitencourt, Reinaldo N. Takahashi

**Affiliations:** ^1^Laboratory of Neuropsychopharmacology, Post-Graduate Program in Health Sciences, University of South Santa Catarina, Tubarão, Brazil; ^2^Laboratory of Psychopharmacology, Department of Pharmacology, Federal University of Santa Catarina, Florianópolis, Brazil

**Keywords:** post-traumatic stress disorder, endocannabinoid system, cannabidiol, aversive memories, fear conditioning paradigm

## Abstract

Post-traumatic stress disorder (PTSD) is characterized by poor adaptation to a traumatic experience. This disorder affects approximately 10% of people at some point in life. Current pharmacological therapies for PTSD have been shown to be inefficient and produce considerable side effects. Since the discovery of the involvement of the endocannabinoid (eCB) system in emotional memory processing, pharmacological manipulation of eCB signaling has become a therapeutic possibility for the treatment of PTSD. Cannabidiol (CBD), a phytocannabinoid constituent of *Cannabis sativa* without the psychoactive effects of Δ^9^-tetrahydrocannabinol, has gained particular attention. Preclinical studies in different rodent behavioral models have shown that CBD can both facilitate the extinction of aversive memories and block their reconsolidation, possibly through potentialization of the eCB system. These results, combined with the currently available pharmacological treatments for PTSD being limited, necessitated testing CBD use with the same therapeutic purpose in humans as well. Indeed, as observed in rodents, recent studies have confirmed the ability of CBD to alter important aspects of aversive memories in humans and promote significant improvements in the symptomatology of PTSD. The goal of this review was to highlight the potential of CBD as a treatment for disorders related to inappropriate retention of aversive memories, by assessing evidence from preclinical to human experimental studies.

## Introduction

Post-traumatic stress disorder (PTSD) is a chronic psychiatric condition that may develop after experiencing a potentially traumatic event. The disorder manifests itself at different levels, through symptoms such as sleep disturbances; changes in cognition (e.g., repeated recall of the event), mood (e.g., depression, anxiety), and emotion (e.g., psychological instability); and reduced social skills. Through the fourth edition of the DSM-IV, post-traumatic stress was classified as an anxiety disorder; however, the latest edition, DSM-V, includes PTSD in a new category called “trauma- and stress-related disorders.”. In this brand-new category, we consider disorders with poor adaptation to a traumatic experience. Maladaptive responses to trauma may trigger, among others, PTSD (Passie et al., 2012; Berardi et al., 2016).

At some point in their lives, approximately 10% of people will be affected by PTSD, resulting in an enormous economic and social impact. This impact is aggravated by the scarcity of psychological and, above all, pharmacological approaches to PTSD treatment (Hidalgo and Davidson, 2000; Yule, 2001; Jurkus et al., 2016). At present, approved treatments for PTSD involve anxiolytics and antidepressants, which are inefficient and have considerable side effects (Berger et al., 2009; Shin et al., 2014; Bernardy and Friedman, 2015).

**FIGURE 1:**
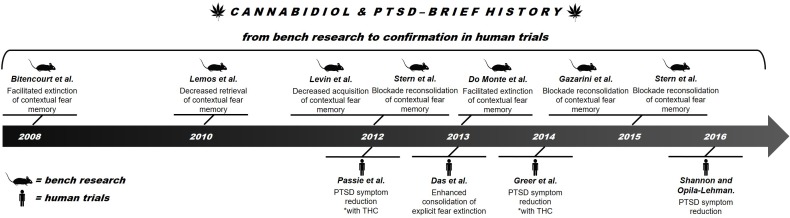
Brief history of advances in research on the use of CBD in PTSD.

The eCB system can provide more efficient and better tolerated alternatives to the standard treatments for PTSD. The eCB system plays an important role in the regulation of emotional behavior and is essential for synaptic processes that determine learning and emotional responses, especially those related to potentially traumatic experiences (Castillo et al., 2012; Riebe et al., 2012). Among the possible alternative approaches, the use of components from *Cannabis sativa* such as CBD is particularly promising. Recent reviews have reported promising results of CBD treatment of several neuropsychiatric disorders, including PTSD (Mechoulam, 2005; Izzo et al., 2009; Passie et al., 2012). What began as a possibility discovered in a study of an animal model of aversive conditioning (Bitencourt et al., 2008) gained strength through results obtained in humans (Das et al., 2013) (see **Figure 1** for a brief history of CBD in PTSD). Because the compound has been proved to be well tolerated by humans, both in overall safety and possible side effects (Bergamaschi et al., 2011), CBD is now considered a new therapeutic possibility for treating PTSD.

This paper reviews the therapeutic potential of CBD in the treatment of PTSD. It starts from the first evidence obtained in animal studies (“bench research”) and proceeds to knowledge gathered in human trials (“confirmation in human trials”).

## Cannabinoids and Trauma-Related Disorder

*Cannabis sativa* contains over 100 compounds called phytocannabinoids. Two of them demonstrate considerable therapeutic potential: Δ9-tetrahydrocannabinol (Δ^9^-THC), considered the main component responsible for the psychoactive effects of the plant, and CBD, the main non-psychotomimetic constituent of *Cannabis* (Adams et al., 1940; Mechoulam and Shvo, 1963; Gaoni and Mechoulam, 1964). CBD constitutes about 40% of the active substances of the plant (Crippa et al., 2009). However, its pharmacological effects are different from, and often even opposite to, those of Δ9-THC, and are not related to the development of tolerance and withdrawal syndrome (Mechoulam et al., 2007; Bergamaschi et al., 2011).

In this context, it is also important to highlight the eCB system, discovered in the 20th century and responsible for a revolution in the understanding of numerous neuropsychological functions related to the modulation of emotional responses (Lutz, 2009). The eCB system comprises two different cannabinoid receptors, their endogenous ligands, and enzymes involved in the synthesis and degradation thereof (Di Marzo, 2009). eCB signaling is distributed throughout the CNS and peripheral tissues, regulating presynaptic release of both excitatory and inhibitory neurotransmitters. Cannabinoid type 1 (CB1) receptors are expressed by peripheral and central neurons, particularly in the central regions known to play important roles in anxiety and aversive learning, such as the amygdala, hippocampus, and cerebral cortex (Childers and Breivogel, 1998). In contrast, CB2 receptors are expressed mostly in immune cells, while also being present in the brain (Van Sickle et al., 2005).

The two major endogenous ligands for CB1 and CB2 receptors are AEA and 2-AG. These eCBs are synthesized on demand, mainly postsynaptically, and act as retrograde messengers regulating the presynaptic release of various neurotransmitters, as mentioned above. AEA acts as a partial agonist of both CB1 and CB2 receptors, with a higher affinity for the former. In the CNS, 2-AG is the most abundant eCB, and non-selectively activates CB1 and CB2 receptors. AEA, 2-AG, and Δ^9^-THC, have been shown to exert their effects mainly through activation of CB1 receptors (Di Marzo, 2009; Castillo et al., 2012). In the case of eCBs, the effects are rapidly terminated through carrier-mediated uptake followed by intracellular enzymatic degradation. AEA and 2-AG are metabolized by monoacylglycerol lipase and FAAH, respectively. The eCBs regulate neuronal activity and plasticity by depolarization-induced suppression of inhibition or excitation (Wilson and Nicoll, 2002). Both phenomena are forms of short-term synaptic plasticity that contribute to the regulation of a number of physiological functions, including memory and emotion. Additionally, eCBs appear to modulate the memory process by changing synaptic plasticity and mediating more persistent forms of synaptic plasticity (e.g., LTP and depression) in several brain areas (Maldonado et al., 2006; Di Marzo, 2009; Sidhpura and Parsons, 2011).

These findings have established the importance of the eCB system in a number of neurophysiological functions and led to an emerging interest in the eCB-mediated modulation of emotionality. The first study to address the role of the eCB system in fear memory, specifically in its extinction, was published at the beginning of the last decade by Marsicano et al. (2002). In this study, the authors showed that genetic deletion of the CB1 receptor or its pharmacological blockade strongly impaired extinction of auditory-conditioned fear and that eCBs were released in the BLA during extinction. This discovery revealed that the eCB system has a central function in the extinction of aversive memories and may therefore be a promising target for the treatment of disorders related to inappropriate retention of such memories (for details see Marsicano et al., 2002). Precisely what processes underlie this function of the eCB system is presently unclear, raising the question whether CBD exerts its effects through a different pathway(s). Previous reports on eCB system involvement in CBD-induced effects have been equivocal. Because the endogenous ligands (e.g., AEA) and Δ^9^-THC act directly on the CB1 receptor, it is possible that some of the effects of CBD are also mediated by this receptor, albeit indirectly. Indeed, CBD may exert its therapeutic effect on PTSD through inhibition of the uptake or enzymatic degradation of eCBs (Bisogno et al., 2001), as suggested by some recent studies (Bitencourt et al., 2008; Do Monte et al., 2013; Elmes et al., 2015; Stern et al., 2015). However, if this is the case, what is the advantage of using CBD over agents that act directly on the CB1 receptor? The answer is simple: fewer complications (specifically, anxiogenic side effects). Agents that target the eCB system directly, such as THC, CB1 agonists, and FAAH inhibitors, have a biphasic effect, in which low doses are anxiolytic, but higher doses can be anxiogenic, in both preclinical models and humans. In contrast, CBD, when administered in acute systemic doses in models of general anxiety, does not cause anxiety even at high doses. However, few studies have examined chronic dosing effects of CBD in models of generalized anxiety, and such studies are needed for the safe long-term use of CBD (Blessing et al., 2015).

Trauma-related disorders may involve dysregulation of the learning process of aversive memories. This process is fundamental for the individual to survive, because through it we avoid potentially dangerous situations without having to respond in a way that damages mental health (Quirk and Mueller, 2008). Consistent with this concept, neural circuits that support fear conditioning are related to circuits that are affected in clinical conditions such as PTSD (Davis and Whalen, 2001). That the available drugs (such as antidepressants and anxiolytics) do not specifically target the memory process may be one of the reasons that pharmacological treatment of PTSD is so difficult (Singewald et al., 2015). Currently approved treatments for PTSD include SSRIs and serotonin/noradrenaline reuptake inhibitors, both with low efficacy (Bernardy and Friedman, 2015). The response rate for SSRIs rarely exceeds 60%, of which less than 30% represents complete remission (Berger et al., 2009). Moreover, the available treatments have considerable side effects, which may limit tolerance or even decrease adherence to the treatment (Shin et al., 2014). In this regard, interventions that act on the eCB system have shown promise since they can affect both the emotional (e.g., relieve PTSD symptoms) and cognitive (e.g., increase the efficiency of psychological approaches) aspects of the disorder (Izzo et al., 2009; Steckler and Risbrough, 2012; Trezza and Campolongo, 2013).

Two important observations led to the consideration of cannabinoids for the treatment of PTSD: (i) patients with PTSD appear to be more likely to smoke *Cannabis*; and (ii) patients with PTSD have increased levels of cannabinoid receptors and reduced peripheral levels of AEA, suggesting that the CB1 receptor upregulation may be a result of low receptor occupancy caused in turn by the deficiency of AEA (Hauer et al., 2013; Hill et al., 2013a; Neumeister, 2013; Neumeister et al., 2013; Loflin et al., 2017). Consistent with these observations, studies have made use of treatment with cannabinoids in animal models of traumatic event exposure to reduce the appearance of PTSD-like behavioral responses. These studies have demonstrated great potential of cannabinoids in the mitigation of maladaptive responses to trauma (for a more detailed review, see Zer-aviv et al., 2016).

When administered after a traumatic situation, cannabinoids may interfere with the acquisition and consolidation of memories of the event, thus mitigating the risk of subsequent symptoms. However, intervention at this stage may be inadvisable because not all people exposed to a traumatic situation will later manifest PTSD. Alternatively, cannabinoids may reduce traumatic memory by affecting its retrieval or reconsolidation, or by stimulating the process of aversive memory extinction. The latter mechanism may hold the most therapeutic promise, especially when taking into account exposure-based psychotherapies, which extinction mechanisms are thought to be engaged (reviewed in Berardi et al., 2016).

Additionally, studies have shown CBD, in its isolated form, to be a constituent of *Cannabis* with enormous therapeutic potential not only for trauma-related disorders, but also for various other psychiatric and neurological disorders (Campos et al., 2012b). Among the advantages of CBD are its high efficacy, lack of psychotomimetic properties or anxiogenic effects caused by eCB transmission activation, inability to induce tolerance and dependence, and safety at high doses both in humans and in animals (Bergamaschi et al., 2011). However, an alternative view must also be considered, according to which the therapeutic effects of *Cannabis* result from the interaction of all the compounds present in the plant (particularly THC and CBD) rather than the isolated action of a single compound. Such interaction, called in the pharmacology the “entourage effect” (Ben-Shabat et al., 1998), still needs to be better studied (for further discussion of the use of the term “entourage effect” on plants effect, see Rosenberg et al., 2015). In this review, we will focus on CBD effects in isolation.

## CBD and PTSD: “From Bench Research…”

The fear-conditioning paradigm has been widely used in animals to better understand the processes of acquisition, consolidation, retrieval, reconsolidation, and extinction of aversive memories (LeDoux, 2000; Maren and Quirk, 2004). Parallels can be drawn between the expression of fear and anxiety in humans (e.g., those suffering from PTSD) to the expression of conditioned fear in animals (Brewin, 2001). Many studies use variations of this model (e.g., contextual fear conditioning) to better understand the effects of CBD on behavioral responses related to the recall of traumatic events. Briefly, this model involves the pairing of a neutral stimulus (called CS) with an aversive US, usually a mild foot shock. After successive rounds of pairing (or, in some cases, a single pairing), the animal learns that the CS precedes the US, leading to a series of physiological (e.g., cardiovascular responses) and behavioral (e.g., freezing) responses (for a more complete description of the fear conditioning paradigm, see Myers and Davis, 2007; Maren, 2008; de Bitencourt et al., 2013).

Intervention in the processes of acquisition and consolidation of aversive memories is not promising, since this approach can only be effective when closely following the traumatic event, that is, when it is not yet possible to know if the event will result in a disorder. However, intervention in the processes of retrieval, reconsolidation, and, especially, extinction may be a more promising alternative. Briefly, when reactivated by re-exposure (retrieval), an aversive memory enters a transitional state, where the original memory trace can be reconsolidated or extinguished. This process may be influenced pharmacologically (e.g., by administration of CBD) in order to block reconsolidation or facilitate extinction. The process involves repeated exposure to the CS without the US, which will lead to the formation of a new, US-free memory trace that will override the old (CS + US) trace and, consequently, cause a decrease in the behavioral and physiological responses related to fear (reviewed in Berardi et al., 2016).

Animal studies have shown that CBD can affect every stage of the process of aversive conditioning. In addition, exposure to traumatic stress is essential for the development of PTSD, and CBD is effective in reducing both the cardiovascular responses and anxiogenic effects caused by stress (Resstel et al., 2006, 2009; Gomes et al., 2011; Campos et al., 2012a, 2013). For example, CBD lowered responses related to trauma when administered before the acquisition (Levin et al., 2012) or retrieval (Lemos et al., 2010) of aversive memories. CBD also proved to be effective in reducing responses to aversive memories by blocking the process of reconsolidation (Stern et al., 2012). However, the most promising alternative, suggested by exposure-based psychotherapies, may be through enhancement of the extinction process by CBD (Bitencourt et al., 2008; Do Monte et al., 2013). Before discussing the facilitating effects of CBD on the extinction of aversive memories, it is necessary to highlight the role of the eCB system in this process.

The process of extinguishing an aversive memory requires the participation of CB1 receptors, which was discovered in a classic study by Marsicano et al. (2002). The authors showed that blocking the action of CB1 receptors, either by pharmacological antagonism or genetic deletion, in previously conditioned mice resulted in strongly impaired short- and long-term extinction in fear-conditioning tests (the function of CB1 receptors in the process of extinction of aversive memories is detailed in Wotjak, 2005; Lutz, 2007). This finding raised a new question: Would potentialization of the eCB system facilitate the extinction process? The answer was not surprising, and several studies were published showing that eCB system potentialization could in fact facilitate fear extinction in different behavioral tasks (Chhatwal et al., 2005; Bitencourt et al., 2008, 2014; de Oliveira Alvares et al., 2008; Pamplona et al., 2008; Abush and Akirav, 2009; Lin et al., 2009). From the answer to the previous question, another arose in Takahashi’s lab – one that would raise CBD as a therapeutic possibility for the treatment of trauma-related disorders: Given that, according to Bisogno et al. (2001), CBD acts through potentialization of the eCB system, could CBD alone also facilitate the extinction of aversive memories? The answer once again was affirmative, and since then studies have shown that CBD can facilitate the extinction of aversive memories not only in animals (Bitencourt et al., 2008; Do Monte et al., 2013), but also in humans (Das et al., 2013). However, it is important to note that some studies suggest that the reduced expression of fear caused by CBD may result mostly from blocked reconsolidation of an aversive memory than its increased extinction (Stern et al., 2012, 2015; Gazarini et al., 2015). Regardless of which stage of aversive memory processing CBD affects, it appears that, at least in animals, this compound interferes with memory processing in a way that potentially mitigates damaging responses.

In addition to the possibility of CBD affecting different processes involved in aversive memory, animal studies also show favorable effects of this compound in the control of other frequent manifestations of PTSD symptomatology, such as sleep disorders. Studies in rats indicate that CBD may contribute to an increase in sleep duration and depth, and a decrease in anxiety responses induced by sleep disturbance (Monti, 1977; Hsiao et al., 2012; Chagas et al., 2013). In the case of anxiety, another frequent manifestation of PTSD symptomatology, therapeutic potential of CBD has also been reported. However, a thorough review of CBD and anxiety lies beyond the scope of this paper. The interested reader may want to see recent reviews by Blessing et al. (2015) and Lee et al. (2017). Even when all evidence from animal studies suggests an enormous therapeutic potential of CBD CBD for treating PTSD symptoms (for a summary, see **Table 1**), it will still be limited if the results are not replicated in humans. Research has also been moving in this direction, as we will see in the next section.

**Table 1:** CBD and PTSD: “from bench research…”.

References in chronological order	Animal/test(s) used	Effective dose/route of administration	Effect	Possible mechanisms of action
Bitencourt et al., 2008.	Rats/Contextual fear conditioning	CBD 2 μg/μl i.c.v.	Facilitated extinction of contextual fear memory	Via CB_1_ receptors
Lemos et al., 2010.	Rats/Contextual fear conditioning	CBD 10 mg/kg i.p. and CBD 30 nmol into the PL PFC.	Decreased retrieval of contextual fear memory	Not shown, but highlights the involvement of the PL PFC
Levin et al., 2012.	Rats/Contextual fear conditioning	CBD 1 mg/kg i.p.	Decreased acquisition of contextual fear memory	Not shown
Stern et al., 2012.	Rats/Contextual fear conditioning	CBD 10 mg/kg i.p.	Blockade reconsolidation of contextual fear memory	Via CB_1_ receptors
Do Monte et al., 2013.	Rats/Contextual fear conditioning	CBD 0.4 μg/side intra-IL cortex	Facilitated extinction of contextual fear memory	Via CB_1_ receptors and highlights the involvement of the IL cortex
Stern et al., 2015.	Rats/Contextual fear conditioning	CBD 1 mg/kg i.p. + THC 0.1 mg/kg i.p.	Blockade reconsolidation of contextual fear memory	Not shown
Gazarini et al., 2015.	Rats/Contextual fear conditioning	CBD 10 mg/kg i.p.	Blockade reconsolidation of contextual fear memory	Not shown


*CBD, cannabidiol; i.c.v., intracerebroventricular; i.p., intraperitoneally; PL PFC, prelimbic prefrontal cortex; IL, infralimbic.*

## CBD and PTSD: “…To Confirmation in Human Trials”

Confirming animal study results in humans is essential for the validation of any strategy that demands a pharmacological therapy. Some studies have shown that, in the case of CBD as a therapeutic alternative for PTSD, this translation is possible (for a summary, see **Table 2**).

**Table 2:** CBD and PTSD: “...to confirmation in human trials.”

References in chronological order	Subjects/test(s) used	Effective dose/route of administration	Effect	Possible mechanisms of action
Passie et al., 2012.	19 year-old male with PTSD (case report)	CBD + THC (cannabis resin from Turkey – 1/1 proportion)/smoked	Patient experienced reduced stress, less involvement with flashbacks and a significant decrease of anxiety.	Not shown
Das et al., 2013.	Healthy humans/Pavlovian fear-conditioning paradigm	CBD 32 mg inhaled	Enhanced consolidation of explicit fear extinction	Not shown
Greer et al., 2014.	80 patients with PTSD	CBD + THC (cannabis – unknown proportion)/smoked	Cannabis (CBD + THC) is associated with PTSD symptom reduction.	Not shown
Shannon and Opila-Lehman, 2016.	10 year-old girl with PTSD (case report)	CBD oil at least 25 mg daily for 5 months/oral capsules	Maintained decrease in anxiety and a steady improvement in the quality and quantity of the patient’s sleep.	Not shown


*CBD, cannabidiol; THC, tetrahydrocannabinol.*

In a study published by Das et al. (2013), CBD increased the consolidation of aversive memory extinction in healthy humans. Using inhaled CBD (at a dose of 32 mg), a study in a model of aversive conditioning showed that the compound caused a reduction in the skin conductance response as well as in the expectation levels for the CS during new exposure. Consistent with results of animal studies, these findings show that CBD may be a pharmacological complement to be used in exposure-based therapy. An important consideration in relation to this study is that CBD facilitated the extinction of aversive conditioning only when administered immediately after, and not before, the process. Therefore, understanding at which moment exposure-based therapy with CBD should start is one of several issues that still need to be resolved (Das et al., 2013).

A case report published in 2016 by Shannon and Opila-Lehman described a 10-year-old child who developed PTSD after being sexually abused before the age of five. The child showed significant relief of the symptomatology using CBD oil. Before the CBD therapy, the child underwent standard pharmacological treatment for the condition, which produced short-lasting partial relief, as well as significant side effects. However, CBD oil (given at a dose of 12–25 mg once a day) appeared to relieve key symptoms, such as anxiety and sleep disturbance, while inducing minimal side effects. Although CBD is considered safe (Bergamaschi et al., 2011), the long-term effects were not evaluated in this study and need to be better elucidated (Shannon and Opila-Lehman, 2016).

In two other studies conducted in patients diagnosed with PTSD (Passie et al., 2012; Greer et al., 2014), chronic use of *Cannabis* significantly decreased the symptoms. However, it is not possible to analyze the proportion of CBD and THC in the plant used by the patients in these studies. Patients with PTSD may use *Cannabis* as a form of self-medication (Hill et al., 2018) in an attempt to reduce their symptoms through the anxiolytic and sedative effects (Bremner et al., 1996; Bonn-Miller et al., 2007), and also to induce sleep (Russo et al., 2007). Recent studies also point to a link between *Cannabis* use, possibly as a form of self-medication, and the occurrence of trauma-related events both in adolescents (Bujarski et al., 2012) and adults (Cougle et al., 2011). The more severe the traumatic experience, the greater the plant consumption (Kevorkian et al., 2015). These findings may reinforce the theory that the entourage effect may be more important to the therapeutic effects of the plant than any single compound used in isolation. To confirm this theory, more studies are required (for a review of *Cannabis* use in people with traumatic experiences, see Zer-aviv et al., 2016).

## Multiple Mechanisms of CBD Action: How Does It Work, Anyway?

The mechanisms of CBD action in behavioral responses related to trauma are still unclear. Understanding the mechanisms underlying CBD action, for example on the expression of aversive memories, is important because a better understanding of this phenomenon may lead to the possibility of more effective interventions in traumatic memories in PTSD. Several mechanisms of action have been proposed to explain the pharmacology of CBD and, as we shall see, they are far from universally accepted.

Within the eCB system, CBD weakly binds to CB1 and CB2 receptors (Pertwee, 2008), and some evidence suggests that it may inhibit both the uptake and hydrolysis (by FAAH) of AEA, an eCB ligand. Thus, CBD may activate CB1 receptors indirectly, by potentiating the eCB system (Bisogno et al., 2001; De Petrocellis et al., 2011; Leweke et al., 2012; Elmes et al., 2015). Based on this assumption and taking into account different studies showing that the activation of CB1 receptors decreases the expression of behaviors related to aversive memories in rats (Chhatwal et al., 2005; Pamplona et al., 2006, 2008; Bitencourt et al., 2008), the action of CBD on such memories may be attributable to indirect potentiation of the eCB system.

A recent review by Hill et al. (2018) proposed that a state of eCB deficiency might represent a stress endophenotype predisposing the individual to the development of trauma-related psychopathology. This work lends further credence to the possibility of CBD enhancing eCB signaling as a possible explanation for the therapeutic effects of CBD and, consequently, its potential to treat PTSD. Furthermore, animal studies have confirmed the importance of the CB1 receptor in mediating the effects of CBD on behavioral responses related to potentially traumatic memories (Bitencourt et al., 2008; Stern et al., 2012, 2015; Do Monte et al., 2013; Gazarini et al., 2015).

However, other research has shown that the answer will not be that simple. In a systematic search of the extant literature for original articles on the molecular pharmacology of CBD, we found a study by Ibeas Bih et al. (2015), which suggested that CBD was unlikely to exert its effects in neurological diseases through modulation of the eCB system. The authors show that CBD can act through 65 discrete, specific molecular targets, including 10 receptors, 32 enzymes, 10 ion channels, and 13 transporters. With regard to the possible modulation of the eCB system, a study published by Massi et al. (2008) showed that CBD stimulated (rather than inhibited, as previously proposed) FAAH, which is involved in the catabolism of AEA; reports of CBD effects on this target are conflicting in the literature. In addition, inhibition of FAAH by CBD *in vitro* is only manifested at high concentrations, which may be difficult to achieve *in vivo*, given the relatively poor bioavailability of CBD (Ibeas Bih et al., 2015). Nevertheless, because FAAH activity appears to be increased by chronic restraint stress in animal models as well as by anxiety-like behaviors (Hill et al., 2013b), FAAH inhibition by CBD appears to us as a possible alternative to explain the CBD effects in aversive memories. In any case, a great deal of caution is needed when interpreting *in vitro* assays and, especially, when extrapolating *in vitro* results to the *in vivo* effects of CBD. Taking into account a possible inhibitory effect of high doses of CBD on the FAAH transporter, it appears likely that incomplete inhibition of FAAH by CBD underlies at least some of its effects *in vivo* (Bisogno et al., 2001; De Petrocellis et al., 2011). This mechanism may be the most promising possibility to explain at a molecular level the inhibitory effects of CBD on behavioral responses related to the recall of traumatic events and it is worth further investigation.

While reports of CBD effects on the eCB system have been contradictory, another molecular target that appears consistent with some of the stress-attenuating effects of CBD may involve serotoninergic transmission via 5-HT receptors (Campos and Guimarães, 2008; Campos et al., 2012a; Gomes et al., 2012). Seven different types of serotoninergic receptors have been identified (5-HT_1-7_), and the 5-HT_1_ class is further subdivided into five other subclasses (5-HT_1A,B,D,E,andF_). Of the latter, 5-HT_1A_ is the main receptor related to CBD effects, with CBD facilitating 5HT_1A_-mediated neurotransmission by acting as an agonist (Russo et al., 2005), promoting anxiolytic effects (Campos and Guimarães, 2008; Gomes et al., 2011; Campos et al., 2012b), mitigating stress responses (Resstel et al., 2009), and, most importantly, reducing the expression of contextual fear conditioning (Gomes et al., 2012). However, controversy regarding the effects of CBD on serotonergic transmission remains. A study by Rock et al. (2012) showed that CBD was not a 5HT_1A_ receptor agonist as originally proposed. In this study, the authors suggested that the 5HT_1A_-mediated effects of CBD might involve allosteric interactions with the receptor binding site or interference with intracellular pathways (Rock et al., 2012). The possible interaction of CBD with the serotonergic receptor, also observed in the eCB system, has not been confirmed *in vivo* (Ibeas Bih et al., 2015).

Another molecular target, still less explored, that may mediate, at least in part, the effects of CBD on the expression of aversive memories, is the adenosinergic system. Domingos et al. (2018) showed that specific pharmacological or genetic blockade of the P2X7R adenosinergic receptor promoted anxiogenic-like effects, along with deficits in extinction learning. It has now been established that the blocking of the eCB system leads to an increase in the expression of fear responses, whereas eCB system stimulation causes a decrease in such responses. Drawing parallels between eCB and adenosinergic signaling, adenosinergic receptor stimulation (direct or indirect) may represent an alternative treatment for trauma-related psychiatric disorders. Moreover, indirect stimulation of the adenosinergic system may explain the effects of CBD on aversive memories. Carrier et al. (2006) showed, also *in vitro*, that CBD decreased the uptake of adenosine and, therefore, might increase endogenous adenosine signaling. Given the precariousness of the extrapolation of *in vitro* results to *in vivo* effects, the potential role of the adenosinergic system in the CBD-induced inhibition of aversive memory expression requires further investigation.

We are still far from reaching a consensus regarding the possibility of other molecular targets mediating the effects of CBD on aversive memories. Precisely for this reason, great care must be taken when interpreting the existing literature as well as proposing new experiments. For a detailed review of the pharmacological mechanisms underlying CBD action, see McPartland et al. (2015), Campos et al. (2016), Lee et al. (2017), and especially Ibeas Bih et al. (2015), who reviewed dozens of potential molecular targets of CBD, questioning its action on the eCB system.

In addition to the behavioral changes induced by treatment with CBD, some of which possibly mediated by the CB1 receptor, studies have also shown that chronic CBD treatment may facilitate neurogenesis in the hippocampus, a structure well known for its important role in processing memories (Wolf et al., 2010; Campos et al., 2013), and that is found reduced in patients with PTSD (Shin et al., 2006). Among the brain areas implicated in the effects of CBD, it is also important to highlight the amygdala, which is hyperactive in patients with PTSD and may be related to the severity of the symptoms (Shin et al., 2006; Patel et al., 2012; Etkin and Wager, 2007). CBD attenuated the level of blood oxygenation in the amygdalae of healthy subjects exposed to different levels of anxiety (Fusar-Poli et al., 2010), and decreased c-fos protein expression in the mouse amygdala (Todd and Arnold, 2016). Reduction in the hyperactivity of the amygdala may also explain, in part, the therapeutic effects of CBD against the symptoms caused by PTSD (Passie et al., 2012). The activity of the mPFC, a brain structure that plays an important role in the effects of CBD on the regulation of aversive responses (Lemos et al., 2010; Do Monte et al., 2013), is also reduced in patients with PTSD (Lanius et al., 2003).

Finally, CBD-induced reduction of trauma-related responses raises a wide spectrum of possibilities involving multiple pharmacological and neural circuit mechanisms. Understanding how these mechanisms work is just one more of the various challenges in the study of cannabinoids as potential treatment for neuropsychiatric disorders.

## Conclusion and Future Perspectives

Human and animal studies suggest that CBD may offer therapeutic benefits for disorders related to inappropriate responses to traumatic memories. The effects of CBD on the different stages of aversive memory processing make this compound a candidate pharmacological adjunct to psychological therapies for PTSD. CBD also shows an action profile with fewer side effects than the pharmacological therapy currently used to treat this type of disorder. In addition, even at high doses, CBD does not show the anxiogenic profile of compounds that directly activate eCB transmission.

However, even in the face of evidence pointing to the modulation of the eCB system, more studies are needed to develop a better understanding of the neurobiological mechanisms involved in CBD responses. Additional controlled studies showing the efficacy of CBD for PTSD in humans are also needed. Although much remains to be discovered about the effects of CBD on PTSD symptoms many steps have already been taken in this direction, which may yield a formulation of CBD for the treatment of patients with trauma and stress-related disorders.

## Author Contributions

RB: first draft. RB and RT: final form of the manuscript.

## Conflict of Interest Statement

The authors declare that the research was conducted in the absence of any commercial or financial relationships that could be construed as a potential conflict of interest.

## Abbreviations

2-AG: 2-arachidonylglycerol
AEA: anandamide
BLA: basolateral amygdala
CBD: cannabidiol
CNS: central nervous system
CS: conditioned stimulus
DSM: Diagnostic and Statistical Manual of Mental Disorders
eCB: endocannabinoid
FAAH: fatty acid amide hydrolase
i.c.v.: intracerebroventricular
i.p.: intraperitoneally
IL: infralimbic
LTD: long-term depression LTD
LTP: long-term potentiation
mPFC: medial prefrontal cortex
PL PFC: prelimbic prefrontal cortex
PTSD: post-traumatic stress disorder
SSRIs: selective serotonin reuptake inhibitors
THC: tetrahydrocannabinol
US: unconditioned stimulus
